# A Single Point Mutation, Asn_16_→Lys, Dictates the Temperature-Sensitivity of the Reovirus tsG453 Mutant

**DOI:** 10.3390/v13020289

**Published:** 2021-02-12

**Authors:** Kathleen K. M. Glover, Danica M. Sutherland, Terence S. Dermody, Kevin M. Coombs

**Affiliations:** 1 Department of Medical Microbiology and Infectious Diseases, Room 543 Basic Medical Sciences Building, 745 Bannatyne Avenue, University of Manitoba, Winnipeg, MB R3E OJ9, Canada; gloverk@myumanitoba.ca; 2 Department of Pediatrics, University of Pittsburgh School of Medicine, 4401 Penn Avenue, Pittsburgh, PA 15224, USA; danica.sutherland@pitt.edu (D.M.S.); terencedermody@chp.edu (T.S.D.); 3 Institute of Infection, Inflammation, and Immunity, UPMC Children’s Hospital of Pittsburgh, 4401 Penn Avenue, Pittsburgh, PA 15224, USA; 4 Department of Microbiology and Molecular Genetics, University of Pittsburgh School of Medicine, 4401 Penn Avenue, Pittsburgh, PA 15224, USA; 5 Manitoba Centre for Proteomics and Systems Biology, 715 McDermot Avenue, University of Manitoba, Winnipeg, MB R3E 3P4, Canada

**Keywords:** reovirus reverse genetics, temperature-sensitive reovirus, site-directed mutagenesis, outer-capsid protein σ3

## Abstract

Studies of conditionally lethal mutants can help delineate the structure-function relationships of biomolecules. Temperature-sensitive (*ts*) mammalian reovirus (MRV) mutants were isolated and characterized many years ago. Two of the most well-defined MRV *ts* mutants are *tsC447*, which contains mutations in the S2 gene encoding viral core protein σ2, and *tsG453*, which contains mutations in the S4 gene encoding major outer-capsid protein σ3. Because many MRV *ts* mutants, including both *tsC447* and *tsG453*, encode multiple amino acid substitutions, the specific amino acid substitutions responsible for the *ts* phenotype are unknown. We used reverse genetics to recover recombinant reoviruses containing the single amino acid polymorphisms present in *ts* mutants *tsC447* and *tsG453* and assessed the recombinant viruses for temperature-sensitivity by efficiency-of-plating assays. Of the three amino acid substitutions in the *tsG453* S4 gene, Asn_16_-Lys was solely responsible for the *tsG453* *ts* phenotype. Additionally, the mutant *ts*C447 Ala_188_-Val mutation did not induce a temperature-sensitive phenotype. This study is the first to employ reverse genetics to identify the dominant amino acid substitutions responsible for the *tsC447* and *tsG453* mutations and relate these substitutions to respective phenotypes. Further studies of other MRV *ts* mutants are warranted to define the sequence polymorphisms responsible for temperature sensitivity.

## 1. Introduction

Mammalian reoviruses (MRVs) are nonenveloped, double-stranded RNA viruses that serve as prototypes of the family *Reoviridae* [1,2]. The MRV genome consists of 10 segments, three large (L1, L2, L3), three medium (M1, M2, M3), and four small (S1, S2, S3, S4) [3]. Other members of this family include rotavirus, which causes viral gastroenteritis in children [4,5,6] and animals, and orbiviruses, which include pathogens of cattle [7,8]. Three main MRV serotypes have been categorized by antibody-mediated neutralization and hemagglutination inhibition and are represented by the prototype strains type 1 Lang (T1L), type 2 Jones (T2J), and type 3 Dearing (T3D). MRV serotypes also are differentiated on the basis of host cell tropism, mechanisms of cell killing, modes of dissemination, and CNS disease [3]. Some MRV strains are reported to possess oncolytic properties against various cancers [9], which is largely mediated by activation of *Ras* signaling pathways [9,10,11]. T3D has undergone numerous clinical trials and possesses marked oncolytic effect against multiple tumors [12].

Conditionally lethal viral mutants have served as useful tools to study various stages of viral replication and assembly [13,14,15,16,17,18,19]. One of the most notable examples is the use of such mutants to elucidate bacteriophage T4 assembly [20]. These mutants have also been used in the design of attenuated viruses for vaccines [21,22]. Several groups generated sets of conditionally lethal temperature-sensitive (*ts*) MRV mutants in the 1960s and 1970s [23,24]. One of the more extensively studied panels of MRV *ts* mutants was isolated after chemical mutagenesis of wild type T3D by Dr. Bernard Fields and was characterized by his colleagues [24,25,26,27,28,29,30]. These mutants were identified by their capacity to form well-defined plaques at a lower “permissive” incubation temperature (generally 30–31 °C). They were also identified by limited replication defined by fewer plaques at a higher “non-permissive/restrictive” temperature (generally 39 °C), whereas wild type T3D forms comparable numbers of plaques at both temperatures. The difference in plaque forming capacity at the restrictive versus permissive temperature is known as efficiency of plating (EOP), and this value is usually <0.03 for many *ts* mutants [19]. To identify the genes responsible for temperature-sensitivity, mutant isolates were crossed with wild-type T1L, and progeny reassortant viruses were screened for EOP [28,29,31,32,33]. Genomic sequencing of some mutants identified specific amino acid residues altered in the *ts* strains [34,35,36,37]. For many of these mutants, the responsible genome segment encodes multiple amino acid substitutions. Therefore, the specific amino acids responsible for the *ts* phenotype are often undefined. For example, the *tsC447* mutant, which fails to assemble core particles at the restrictive temperature, contains three mutations in the S2 gene that encodes major core scaffold protein σ2; Ala_188_→Val, Ala_323_→Val, and Asn_383_→Asp [35]. Passaging studies of this mutant, conducted at the restrictive temperature, led to the rescue of a small number of revertant viruses. Sequence determinations of these revertant S2 genes suggest that the Asn_383_→Asp alteration in *tsC447* is responsible for the *ts* phenotype, because reversion at this site allowed wild-type levels of replication at the restrictive temperature [38]. Similarly, the *tsG453* mutant, which only forms core-like particles at the non-permissive temperature [36], contains three mutations in the S4 gene that encodes major outer-capsid protein σ3; Asn_16_→Lys, Met_141_→Ile, and Glu_229_→Asp [36,37]. Mutant *tsG453* σ3 does not associate with major outer-capsid protein µ1 [37], supporting a model in which inner core particles are incapable of obtaining an outer capsid and do not mature to virions at restrictive temperatures.

Kobayashi et al. [39] developed a reverse genetics system to introduce changes into specific MRV genome segments. Altered viruses are recovered following transfection of susceptible cells with 4 to 10 plasmids that encode cDNA for all 10 MRV RNA genome segments under transcriptional control of the bacteriophage T7 RNA polymerase promoter and are fused at the 3′ terminus with hepatitis delta virus ribozyme sequences [40]. We used this reverse genetics system to construct virus clones containing each of the individual *tsC447* and *tsG453* mutations to identify the specific amino acid residues responsible for the phenotype of each mutant. We recovered viral clones containing each of the individual *tsG453* mutations, and EOP values of each indicated that the Asn_16_→Lys substitution was solely responsible for the *tsG453 ts* phenotype. We were unable to recover all of the *tsC447* mutations individually, but the isolates examined suggest that Asn_383_→Asp is responsible for the *tsC447 ts* phenotype, in agreement with prior reversion analyses [38].

## 2. Materials and Methods

### 2.1. Cells

Murine L929 fibroblasts were maintained in Joklik-modified Eagle’s minimal essential medium (J-MEM) supplemented to contain 5% fetal bovine serum (FBS), 2 mM L-glutamine, 100 U penicillin G/mL, 100 µg streptomycin/mL, and 0.25 µg amphotericin B/mL (Gibco/Life Technologies, Grand Island, New York). Baby hamster kidney cells engineered to stably express T7 RNA polymerase (BHK-T7) cells were maintained in Dulbecco’s modified Eagle medium (Gibco) supplemented to contain 5% FBS, 2 mM l-glutamine, 2% MEM non-essential amino acid solution (Gibco), and 1 mg/mL geneticin (Invitrogen, Waltham, MA, USA).

### 2.2. Plasmids

Seven plasmids were used that contained cDNAs corresponding to the 10 reovirus T3D gene segments cloned into pT7-cDNA, as described [41]. *E. coli* DH5α competent cells were transformed with T3D L1/M2, T3D L2/M3, T3D L3/S3, T3D M1, T3D S1, T3D S2, or T3D S4 plasmids. *E. coli* were amplified on Luria agar or in Luria broth supplemented with ampicillin, and plasmids were isolated using plasmid purification kits (Qiagen, Hilden, Germany).

### 2.3. Primer Design for ts Mutants

The QuikChange Primer Design tool (https://www.agilent.com/store/primerDesignProgram.jsp) was used to design primer sets to introduce each mutation in the *ts* S2 gene of *tsC447* and the *ts* S4 gene of *tsG453* into the wild-type T3D S2 and S4 gene plasmids, respectively. Corresponding primer sequences (listed 5′ to 3′) are:

T3D S2 A188V F-CAATGTGTATGCAATCTCTACAAACGTGTGCCCGAAATA
T3D S2 A188V R-TATTTCGGGCACACGTTTGTAGAGATTGCATACACATTG
T3D S2 A323V F-CATGCAATTGGTTACCAACTCTACCAGTCCAGCCA
T3D S2 A323V R-TGGCTGGACTGGTAGAGTTGGTAACCAATTGCATG
T3D S2 N383D F-GGATGAGCCTGACTATATTGATCGTCTTCTCTCGCC
T3D S2 N383D R-GGCGAGAGAAGACGATCAATATAGTCAGGCTCATCC
T3D S2 N383S F-GGATGAGCCTGACTCTATTGATCGTCTTCTCTCGCC
T3D S2 N383S R-GGCGAGAGAAGACGATCAATATAGTCAGGCTCATCC
T3D S4 N16K F-CCTTCAAAAGCGTTCTTAATCAAGTCCACGACCTGAT
T3D S4 N16K R-ATCAGGTCGTGGACTTGATTAAGAACGCTTTTGAAGG
T3D S4 M141I F-CAACTTGAGTGTATTGATCTAAATATTGAATTTGGGTCAACCTGAAG
T3D S4 M141I R-CTTCAGGTTGACCCAAATTCAATATTTAGATCAATACACTCAAGTTG
T3D S4 E229D F-CCCTTCGATGGATCATGATCCAGCTCAGAGTAATC
T3D S4 E229D R-GATTACTCTGAGCTGGATCATGATCCATCGAAGGG

### 2.4. Reovirus Plasmid Mutagenesis

PCR-based site-directed mutagenesis was used to introduce single or multiple mutations into the plasmids encoding wild-type genes. All PCR product sizes were confirmed by agarose gel electrophoresis. PCR products were digested with Dpn1 to remove template DNA and transformed into DH5α cells. Mutagenized and wild-type plasmid DNA was purified from DH5α and sequence-confirmed using Sanger sequencing and Serial Cloner 2.6.1 software.

### 2.5. Reovirus Reverse Genetics

BHK-T7 cells were passaged overnight in Dulbecco-modified Eagle medium (DMEM) medium supplemented to contain geneticin to maintain efficient expression of the T7 RNA polymerase, which mediates transcription of the reovirus cDNAs. Prior to transfection, cells were supplemented with geneticin-free DMEM medium and maintained at 37 °C. Transfection mixtures consisted of OptiMEM, TransIT-LTI, and 2.53 µg of each of the seven reverse genetics plasmids. Transfection mixtures were incubated at room temperature for 30 min and added dropwise to cells. Cells were incubated at 34.5 °C to allow rescue of the *ts* mutant virus. Positive control transfections were included that contained wild-type T3D S2 for *tsC447* and wild-type T3D S4 for *tsG453*. Cells were observed for a maximum of 5 days for visible cytopathic effects. Cell lysates were prepared using two freeze-thaw cycles and stored at 4 °C for no more than a few days, or −80 °C for longer intervals. Fluorescent focus unit assays (FFUs) were conducted by inoculating L929 cells with lysates from transformed cells, followed by staining with polyclonal rabbit anti-reovirus antiserum (prepared by inoculating New Zealand white rabbits with reovirus strains T1L or T3D). Sera from T1L- and T3D-inoculated rabbits were mixed 1:1 (*vol*/*vol*), and nonspecific antibodies were depleted using cross-adsorption on methanol-fixed L929 cells. A fluorophore-conjugated, goat anti-rabbit secondary antibody was used to visualize reovirus infection.

### 2.6. Sanger Sequencing for Rescued Virus

L929 cells were inoculated with transformed cell lysates, and the resultant virus was plaque-purified and passaged in L929 cells at 34.5 °C (permissive temperature) to recover passage 1 (P1) and passage 2 (P2) viral stocks. Total RNA was purified from P2 cell lysates by phenol:chloroform extraction, and reovirus S2 or S4 RNA was converted to cDNA using primers specific for the termini of the S2 or S4 gene segments and the Qiagen OneStep RT-PCR kit according to the manufacturer’s instructions (Qiagen OneStep RT-PCR Handbook, 10/2012). Amplification primers were:

T3D S2 F-GCTATTCGCTGGTCAGTTAT
T3D S2 R-ATGAATGTGTGGTCAGTCGT
T3D S4 F-CGTTGTCGCAATGGAGGTGTGCTTGC
T3D S4 R-AGCCTGTCCCACGTCACACC

Thermal cycler conditions were maintained as follows: reverse transcription at 50 °C for 30 min; initial PCR activation step at 95 °C for 15 min; and 34 cycles of 3-step cycling that included denaturation at 94 °C for 60 s, annealing at 56 °C for 60 s, extension at 72 °C for 150 s, and final extension at 72 °C for 60 s. The sequence of amplified cDNA was determined by the Sanger method using amplification primers (listed above). Sequence data were analyzed using Sequence Scanner (Applied Biosystems, Foster City, California) and Serial Cloner.

### 2.7. Reovirus Efficiency of Plating (EOP) Assays

Reovirus EOP assays were conducted as described [19]. Briefly, sets of confluent monolayers of L929 cells in 6-well plates were inoculated with 10-fold serial dilutions of each P2 virus stock. After adsorption, inoculated cells were overlaid with a 1:1 mixture of 2% Difco-Bacto agar and completed plaque assay medium (2× Medium-199 supplemented to contain 6% FBS, 4mM l-glutamine, 200 U penicillin G/mL, 200 µg streptomycin/mL, and 0.5 µg amphotericin B/mL). Plates were incubated at 39 or 34.5 °C for a total of 5 or 8 days, respectively. Plates incubated at 34.5 °C were supplemented with additional agar:medium mixture on day 3 post-infection. At 5 or 8 days post-inoculation, monolayers were fixed with 2.5% formalin in PBS, and agar plugs were removed. Monolayers were re-fixed and stained with crystal violet. Plaques were photographed, and viral titers at different temperatures were compared to determine EOP.

### 2.8. 3-Dimensional Protein Analyses

The coordinates of the asymmetric unit of the reovirus core (PDB 1EJ6) and the reovirus μ1/σ3 heterohexamer (PDB 1JMU) were obtained from the Research Collaboratory for Structural Bioinformatics (RCSB) PDB protein databank (http://www.rcsb.org (accessed on 27 December 2020)) and exported to the UCSF Chimera program (version 1.13.1) to visualize reovirus protein structures. Relevant amino acids were identified and colored within the program.

## 3. Results

### 3.1. Reverse Genetics Rescue of Infectious Reovirus Clones Containing Individual and Sets of tsC447 and tsG453 Temperature-Sensitive Alterations

**Figure 1:**
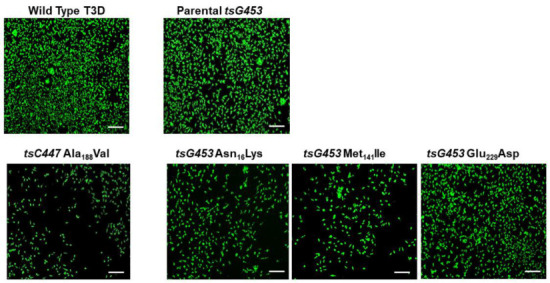
Confirmation of recovery of wild-type T3D and five mutant isolates. Representative images of a fluorescent focus unit assay are shown. L929 cells were adsorbed with BHK-T7 cell lysates from reverse genetics experiments involving the indicated mutants. At 20 hpi, cells were fixed and stained with a rabbit polyclonal anti-reovirus antiserum and a fluorophore-conjugated, goat anti-rabbit secondary antibody to visualize reovirus infection. Scale bars are 200 µm.

Plasmids containing each individual tsC447 S2 or tsG453 S4 mutation, or plasmids containing all three mutations of each *ts* mutant, were combined with plasmids containing the other nine reovirus genes and transfected into BHK-T7 cells. Rescue cell lysates were inoculated onto indicator cells and infection was monitored by fluorescent focus unit assay to confirm recovery of infectious reovirus (Figure 1). We recovered isolates containing each of the individual tsG453 polymorphisms (Asn_16_→Lys, Met_141_→Ile, and Glu_229_→Asp) and an isolate that contained all three parental tsG453 polymorphisms. We also recovered the S2 Ala_188_→Val single-mutant isolate. However, despite numerous attempts, we were unable to recover a plaque-forming virus containing either of the individual tsC447 Ala_323_-Val and Asn_383_-Asp polymorphisms.

**Figure 2:**
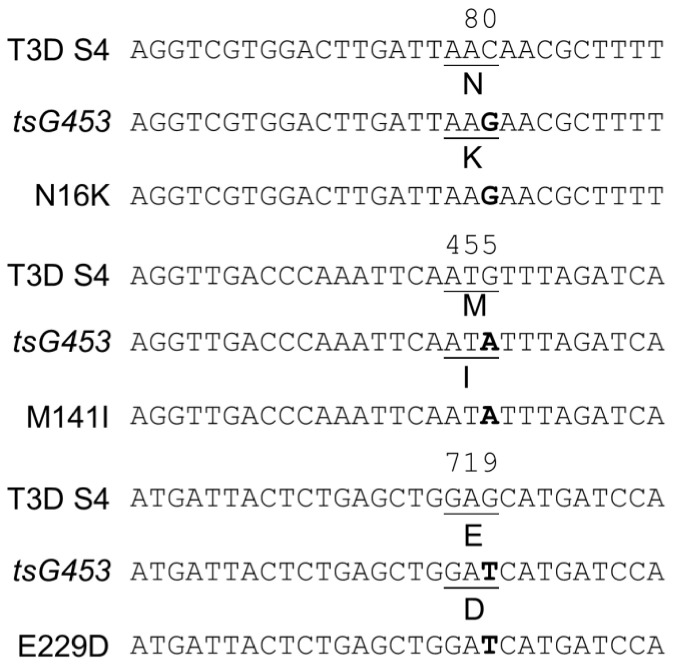
Sanger sequence confirmation of parental *tsG453* and isolates containing each individual mutation. The top line depicts the wild-type T3D S4 nucleotide sequence and relevant σ3 amino acid residue. The second line depicts the corresponding sequence in the *ts*G453 mutant [36,37]. The bottom sequence line depicts the corresponding sequence in the indicated rescued isolate. Data for the T3D S2 gene (*tsC447*) are not shown because only a single recombinant isolate sequence was recovered.

Viral RNA was extracted from P2 stocks of each isolate, and sequences of purified cDNA were determined using the Sanger technique to confirm the presence of each mutation and the absence of any additional mutations. Sequencing confirmed the tsC447 Ala_188_-Val mutation and the tsG453 Asn_16_→Lys, Met_141_→Ile, and Glu_229_→Asp mutations in the expected isolates (Figure 2). Moreover, no additional mutations were observed in the altered genes of interest for these isolates.

### 3.2. Efficiency of Plating Values of Various Rescued Clones

**Figure 3:**
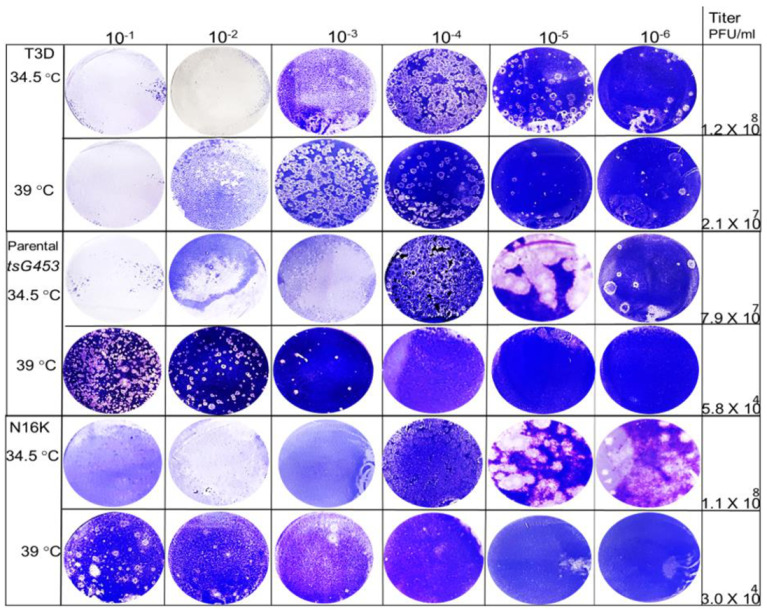
Viral plaques produced by various rescued isolates at 34.5 and 39 °C. Ten-fold serial dilutions of P2 stocks for each isolate were adsorbed to mouse L929 monolayers and incubated for 5 days (39 °C) or 8 days (34.5 °C). Cell monolayers were fixed and stained with crystal violet.

We tested the temperature sensitivity of each rescued isolate to define the amino acid substitution(s) responsible for the respective mutant phenotypes. Because reovirus *ts* mutants replicate comparably at both 31 and 34.5 °C [19], we used 34.5 °C as the representative permissive temperature [19] and 39 °C as the non-permissive temperature. Therefore, we determined the capacity of each isolate to form plaques at each temperature and calculated the resulting EOP values. All isolates produced large, well-circumscribed plaques at 34.5 °C (Figure 3).

**Figure 4:**
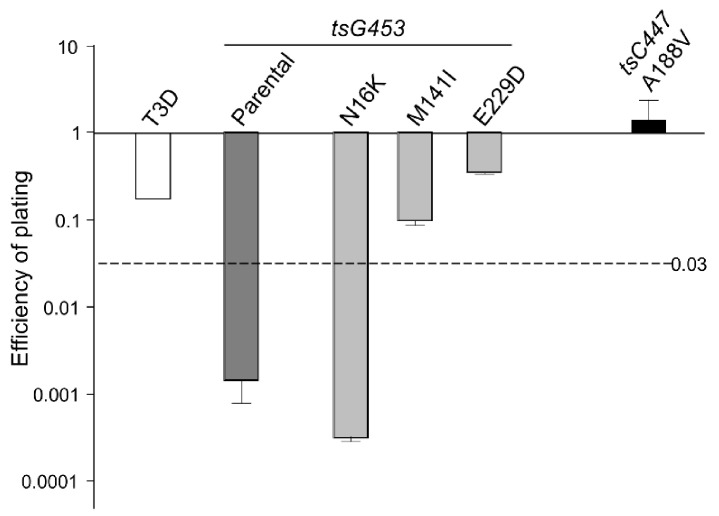
Efficiency of plating (EOP) of rescued virus isolates. EOP values were calculated by dividing the titer produced by the viruses shown at the restrictive incubation temperature of 39 °C by the titer at the permissive temperature of 34.5 °C. *n* = 3; Error bars = SEM.

At higher temperatures, plaques were also formed efficiently by the A188V, M141I, and E229D mutants at dilutions greater than 10^−4^. In contrast, the parental tsG453 and N16K mutants formed smaller-sized plaques at a higher temperature than those formed at 34.5 °C and only produced plaques at dilutions less than 10^−3^, indicating a potential *ts* defect (Figure 3). Indeed, the calculated 39 °C/34.5 °C EOP values indicate that only parental tsG453 and N16K had values significantly below 0.03 (Figure 4), indicating a *ts* phenotype.

## 4. Discussion

**Figure 5:**
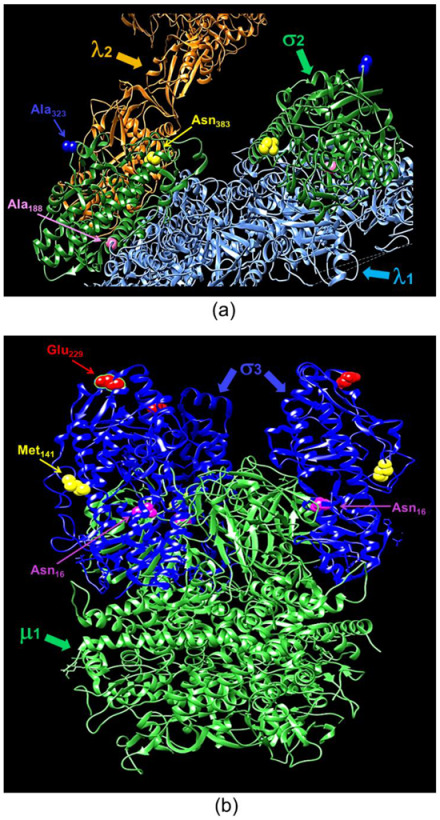
Ribbon tracings of the reovirus σ2 and σ3 proteins with annotated point mutation sites. (**a**) A mammalian reoviruses (MRV) core with T1L σ2 depicted in green. Residues altered in *tsC447* are indicated. Ala_188_ and Asn_383_ are located near λ1 (light blue), and A_323_ is located near λ2 (orange). (**b**) A heterohexamer assembly of T1L σ3 and μ1. Locations of *tsG453* mutations are indicated on each of the three σ3 monomers (in blue). Asn_16_ is located near the σ3 interface with μ1 (green), Met_141_ is located adjacent to several unstructured loops on the lateral surface of σ3, and Glu_229_ is located on the apical surface of σ3. A lysine substitution at Asn_16_ is postulated to alter σ3 contacts with the neighboring μ1 protein. Ribbon tracing images were produced using PDB 1EJ6 (λ1-λ2-σ2) and PDB 1JMU (μ1-σ3) and UCSF Chimera.

The elucidation of macromolecular structure-function relationships has been aided by studies of conditionally lethal mutants. For example, many of the molecular steps in the assembly of bacteriophage T4 [20] and of mammalian orthoreoviruses [19,33,37] have been defined by analyses of such mutants. Two MRV *ts* mutants that have been studied extensively are the *tsC447* mutant with lesions in the MRV S2 gene that encodes the σ2 core protein and the *tsG453* mutant with lesions in the MRV S4 gene that encodes the σ3 outer-capsid protein [29]. The σ2 protein, present in 150 copies per virion [42], serves as a clamp to stabilize the λ1 core shell [43]. At the non-permissive temperature of 39 °C, the *tsC447* mutant produces less RNA and fails to assemble core-like particles, yielding thin empty-shell structures [44]. The *tsC447* S2 gene contains three polymorphisms compared to the wild-type T3D S2 sequence [35]. Identification of these polymorphism sites within the σ2 atomic structure demonstrates that the altered amino acids are located in different regions of the protein (Figure 5a). Both Ala_188_ and Asn_383_ are located near λ1; therefore, mutations at either site, or both, could explain the *tsC447* phenotype. Successful recovery of the Ala_188_→Val isolate and its lack of temperature-sensitivity (Figure 4) indicate that the Ala_188_→Val alteration does not contribute to the *ts* phenotype of *tsC447*. Thus, either the Ala_323_→Val or Asn_383_→Asp, or both, mutations are responsible for the *tsC447 ts* phenotype. Ala_323_ is located at the periphery of the σ2 protein and, based on its position, is unlikely to interact with any other core proteins. It may interact with outer-capsid protein μ1, but this would likely not explain the failure of this mutant to produce a core particle, in apparent agreement with reversion analyses that indicated Asn_383_→Asp was solely responsible [38]. Unfortunately, the inability to rescue clones containing the individual Ala_323_→Val or Asn_383_→Asp mutations, despite repeated attempts, prevented us from further testing the roles of these individual amino acids in σ2 structure-function. It is possible that each of these individual polymorphisms, in the absence of the others, results in a lethal phenotype that prevented their rescue. Alternatively, RNA secondary structure negatively impedes translation of proteins by slowing or blocking the initiation and movement of ribosomes along the mRNA [45,46], and might explain our inability to rescue the individual Ala_323_→Val or Asn_383_→Asp mutations. However, RNA secondary structure predictions of the corresponding S2 genome segments of the corresponding Ala_323_→Val or Asn_383_→Asp mutants [47,48] did not reveal any noticeable differences in the predicted folding patterns compared to the parental T3D or tsC447 S2 genome folding patterns.

**Figure 6:**
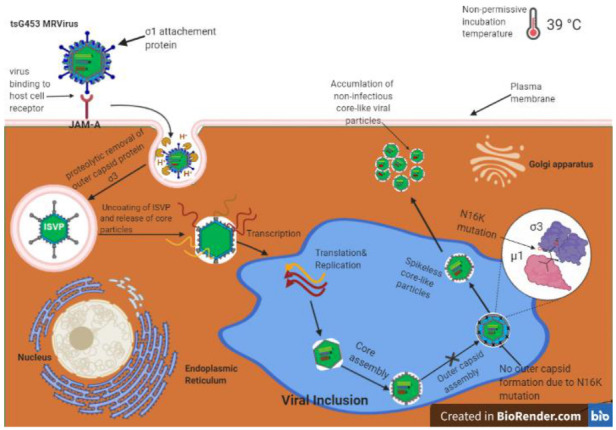
Model for reovirus *tsG453* mutant capsid assembly. Reovirus binds to the receptor junction adhesion molecule A (JAM-A; top left), undergoes internalization, and outer-capsid proteins are removed to yield transcriptionally active core particles (center). Viral mRNAs are translated to produce viral proteins and also serve as templates for replication of progeny (−)-sense RNA. Viral proteins encapsidate viral RNA to produce progeny particles in viral inclusions (blue area in center). At the non-permissive temperature of 39 °C, progeny core-like particles are produced. However, the σ3-N16K mutation prevents σ3 from forming complexes with outer-capsid protein µ1. Therefore, core-like particles accumulate in cells, and viral replication is inhibited.

The MRV σ3 protein, present in 600 copies in complex with the μ1 protein, forms the outermost shell of the virion [42,49]. It is the first protein removed by proteolysis during viral entry into cells [50,51,52,53] or in the intestinal lumen [54,55]. This protein has numerous functions during viral replication, including suppression of protein kinase R (PKR) activation [56,57]. The *tsG453* mutant produces comparable levels of viral protein and RNA during infections at permissive and non-permissive temperatures [26,58], but this mutant produces only core-like particles [36]. The failure of this mutant to assemble intact virions has been attributed to lack of mutant σ3 protein association with the μ1 protein at restrictive temperatures [37]. Sequencing of the *tsG453* mutant S4 gene identified three polymorphisms compared to wild-type T3D S4 [36,37]. Identification of these polymorphism sites within the σ3 atomic structure demonstrates that the altered amino acids are located in different regions of the protein (Figure 5b). Met_141_ and Glu_229_ are located on the periphery of the σ3 protein and, based on their positions, are unlikely to interact with any other viral proteins. Furthermore, EOP analyses (Figure 4) indicate that neither the M141I nor the E229D alterations contribute to the mutant phenotype. Of the isolates tested, only the parental rescued mutant with all three amino acid substitutions and the isolate containing the Asn_16_→Lys alteration had EOP values substantially below 0.03, indicating that this single amino acid residue is responsible for the *ts* phenotype of *tsG453*. Asn_16_ is located at an interface with μ1; therefore, it is possible that a change from asparagine to a substantially more basic, extended lysine residue could perturb the σ3 protein so that at the non-permissive temperature σ3 is unable to interact stably with μ1 [37]. The failure of the *tsG453* mutant to assemble beyond a core-like particle at the non-permissive temperature (Figure 6), combined with the apparent incapacity of mutant σ3 to interact with μ1 [37], suggest that σ3/μ1 interactions and formation of the heterohexameric complex are prerequisites for assembly of the reovirus outer capsid.

## 5. Conclusions

In conclusion, this study is the first to employ reverse genetics to precisely define the amino acid polymorphisms responsible for the *ts* phenotype in at least one of the Fields’ panel of MRV *ts* mutants. Several *ts* isolates remain for which there is no information about the amino acid alternations that confer the *ts* phenotype. In addition, further studies using the mutants studied here should be conducted to determine what effects, if any, alter the atomic structures of the relevant proteins, thereby better delineating the functions of these proteins.

## Data Availability

All data present within manuscript.
